# Genomic Insights into Methicillin-Resistant Staphylococcus aureus *spa* Type t899 Isolates Belonging to Different Sequence Types

**DOI:** 10.1128/AEM.01994-20

**Published:** 2021-02-26

**Authors:** Henok Ayalew Tegegne, Ivana Koláčková, Martina Florianová, Pierre Wattiau, Tereza Gelbíčová, Cécile Boland, Jean-Yves Madec, Marisa Haenni, Renáta Karpíšková

**Affiliations:** a Department of Bacteriology, Veterinary Research Institute, Brno, Czech Republic; b Faculty of Veterinary Hygiene and Ecology, University of Veterinary and Pharmaceutical Sciences Brno, Brno, Czech Republic; c Veterinary Bacteriology, Sciensano, Brussels, Belgium; d Unité Antibiorésistance et Virulence Bactériennes, University of Lyon-ANSES, Lyon, France; University of Helsinki

**Keywords:** MRSA, t899, spa type, MLST, cgMLST, whole-genome sequencing

## Abstract

Methicillin-resistant Staphylococcus aureus (MRSA) presenting *spa* type t899 is commonly associated with sequence type 9 (ST9) but is also increasingly linked to ST398. This study provides genomic insight into the diversity of t899 isolates using core genome multilocus sequence typing (cgMLST), single nucleotide polymorphism (SNP)-based phylogeny, and the description of selected antimicrobial resistance and virulence markers. The SNP-based phylogenic tree showed that isolates sharing the same *spa* type (t899) but different STs highly diverged in their core and accessory genomes, revealing discriminant antimicrobial resistance (AMR) and virulence markers. Our results highlighted the idea that in a surveillance context where only *spa* typing is used, an additional multiplex PCR for the detection of the *tet*(M), *sak*, and *seg* genes would be valuable in helping distinguish ST9 from ST398 isolates on a routine basis.

**IMPORTANCE** This study showed the genetic diversity and population structure of S. aureus presenting the same *spa* type, t899, but belonging to different STs. Our findings revealed that these isolates vary deeply in their core and accessory genomes, contrary to what is regularly inferred from studies using *spa* typing only. Given that identical *spa* types can be associated with different STs and that *spa* typing only is not appropriate for S. aureus isolates that have undergone major recombination events which include the passage of the *spa* gene (such as in t899-positive MRSA), the combination of both MLST and *spa* typing methods is recommended. However, *spa* typing alone is still largely used in surveillance studies and basic characterization. Our data suggest that additional markers, such as *tet*(M), *sak*, and *seg* genes, could be implemented in an easy and inexpensive manner in order to identify S. aureus lineages with a higher accuracy.

## Abstract (precis)

This study showed the genetic diversity and population structure of S. aureus presenting the same *spa* type, t899, but belonging to different STs. Our findings revealed that these isolates vary deeply in their core and accessory genomes, contrary to what is regularly inferred from studies using *spa* typing only.

## INTRODUCTION

Livestock-associated methicillin-resistant Staphylococcus aureus (LA-MRSA) was first described in 2005 (1, 2) and rapidly gained importance because of its capacity to colonize and infect humans, particularly pig farmers. Clonal complex 398 (CC398) has been referred to as the predominant LA-MRSA lineage in Europe (3, 4), but other lineages, including CC9 and CC5, have also been detected (3–7).

*spa* type t899 originally belongs to CC9 (sequence type 9 [ST9]), the predominant LA-MRSA genotype reported in Asia, and was identified in pigs and their associated human workers (8). More recently, *spa* type t899 was also reported in a CC9/CC398 hybrid strain in Europe (9, 10). This hybrid is a unique genotype with a CC398 chromosomal backbone and smaller CC9 region with a *spa* gene (9). CC9 differs from the European pig-associated CC398 with regard to clonal type, staphylococcal cassette chromosome *mec* element (SCC*mec*) content, and resistance profile (4).

CC398 (ST398) isolates belonging to *spa* type t899 have received specific attention because they cause sporadic illness in humans (9, 11–13). This lineage notably harbors the staphylococcal complement inhibitor (*scn*), staphylokinase (*sak*), and chemotaxis inhibitory protein (*chp*) genes associated with the ϕSa3 immune evasion cluster (IEC), allowing adaptation to the human host (10). *spa* types t011 and t034 (which display close tandem repeat successions) are still the main types associated with CC398, but t899 is increasingly described in the literature both in human and veterinary medicine (14–19).

The CC9/CC398 hybrid strain has been identified from livestock (9, 10) and related personnel (11, 20) in several European countries. Furthermore, two CC398 LA-MRSA *spa* type t899 isolates were recently reported as incidental findings during a clinical investigation from turkey and pheasant in the United Kingdom (10, 21). These two isolates were shown to belong to the CC9/C398 hybrid genotype and were quite similar to the clone that was reported from continental Europe (10, 13, 22). Interestingly, t899 has been increasingly associated with several single-locus variants (SLVs) of CC398 and CC9 (16–18).

Overall, the occurrence of the same *spa* types in distant lineages has been reported, resulting from either convergent evolution or genetic recombination (23, 24). As an example, CC239 MRSA is a hybrid strain of CC30 (founder, ST30) and CC8 (founder, ST8) (23). ST34 and ST42 backgrounds have also been suggested to be of hybrid origin (24). Recently, t304 isolates belonging to ST6, ST1649, ST8, and ST4290 were reported by Bartels et al. (25).

Since *spa* typing is still largely used as the unique typing method, for example in large surveillance studies and in low-income countries, our aim was to characterize t899 isolates using single nucleotide polymorphism (SNP)-based phylogeny on publicly available genomic data and associated metadata in order to identify markers that could be implemented in an easy and inexpensive manner in order to identify LA-MRSA lineages with a higher accuracy.

## RESULTS

**FIG 1:**
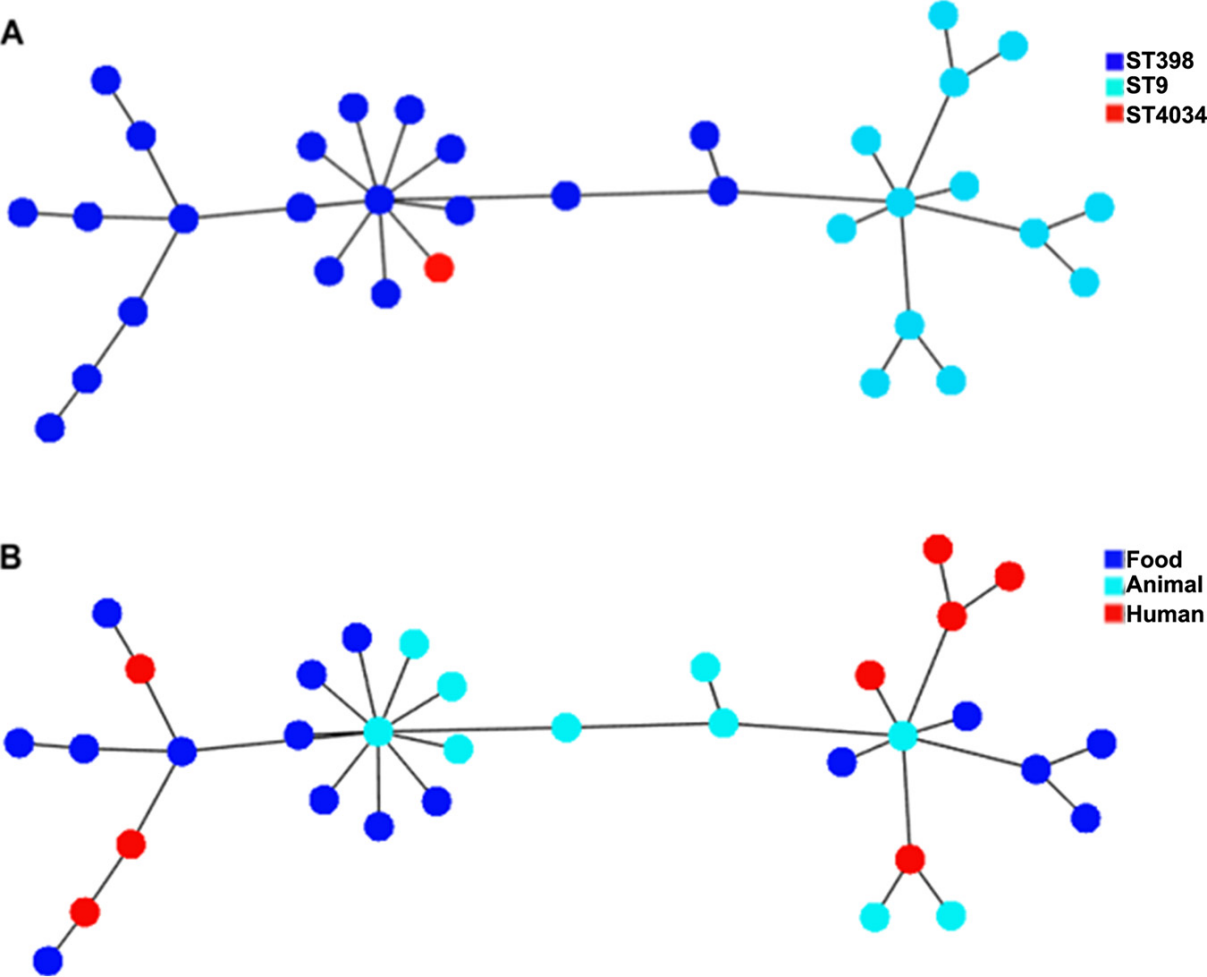
Minimum spanning tree based on the concatenated core genome of 34 S. aureus t899 strains with color annotation based on (A) MLST and (B) matrix. Visualization was realized using PHYLOViZ.

**FIG 2:**
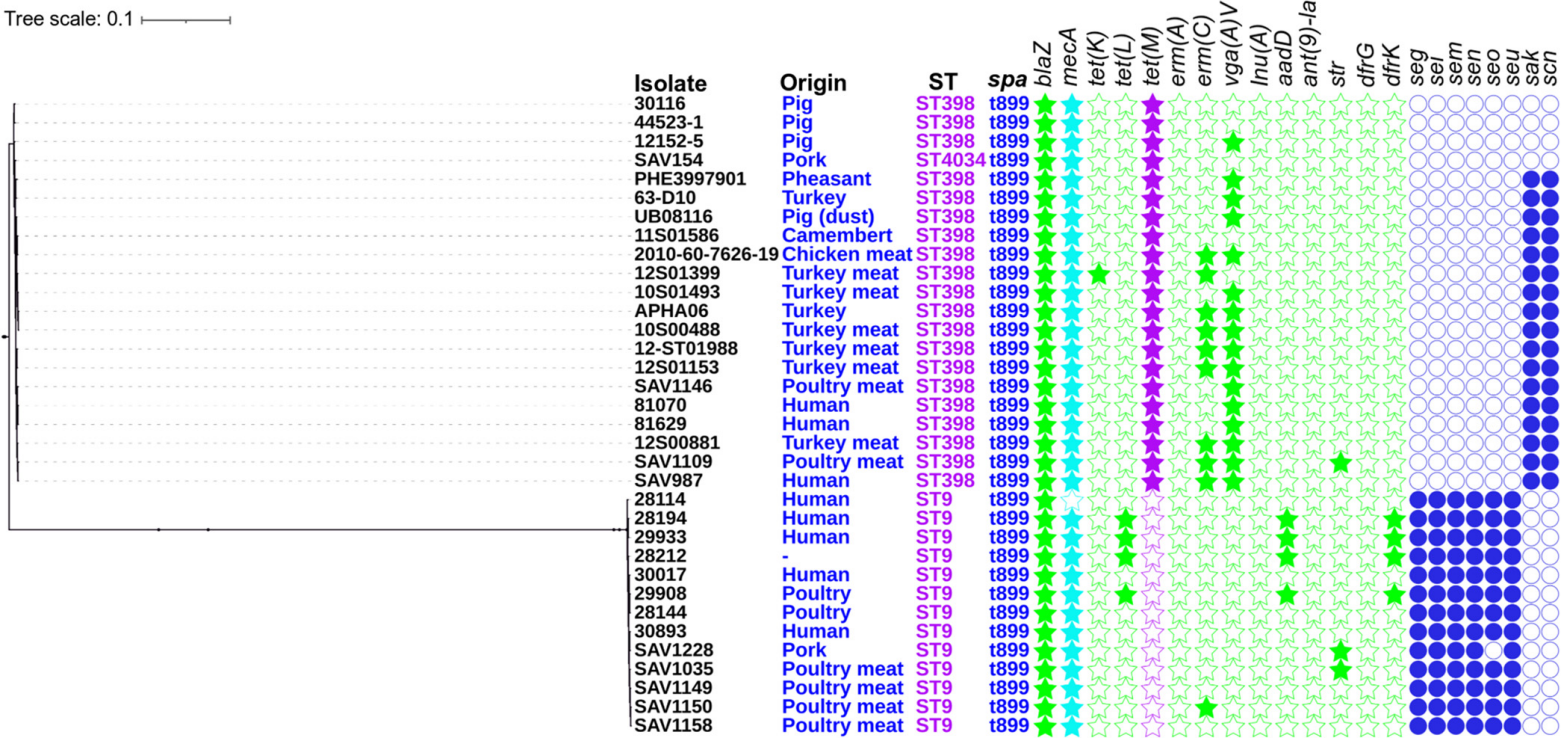
Maximum-likelihood phylogenetic tree based on SNP analyses of 34 S. aureus t899 strains obtained by CSI Phylogeny from the Center for Genomic Epidemiology web-based platform. Sequence type (ST), *spa* type (*spa*), isolate origin, the presence of antimicrobial resistance, and virulence markers are indicated. The filled stars and circles indicate confirmed antimicrobial resistance and virulence markers, respectively, as extracted from ResFinder and Virulence Finder. Visualization was realized using iTOL. Isolates with SAV numbers are unique to this study. Genes encoding resistance to beta-lactams (*bla*Z, *mecA*), tetracycline [*tet*(K), *tet*(M), *tet*(L)], macrolide-lincosamide-streptogramin B [*erm*(A), *erm*(C)], pleuromutilin-lincosamide-streptogramin A [*vga*(A)V], lincosamides [*lnu*(A)], aminoglycosides [*aadD*, *ant*(9)-la, *str*], and trimethoprim (*dfrK*, *dfrG*) and those encoding staphylococcal enterotoxins (*seg*, *sei*, *sem*, *sen*, *seo*, *seu*), staphylokinase (*sak*), and staphylococcal complement inhibitor (*scn*) are shown.

Thirty-four LA-MRSA genomes of t899 isolates were analyzed, of which 20 belonged to ST398, 13 to ST9, and 1 to ST4034 (a single-locus variant [SLV] of ST398, differing by one substitution [A_294_T] in the *arcC* gene). Metadata associated with the selected isolates were recorded (Table 1). All t899 isolates harbored the *mecA* gene on a SCC*mec* IVa(2B) element, except for two which presented either the SCC*mec* V element or an undefined cassette. Both the spanning tree and the SNP-based phylogenetic tree (Fig. 1B and 2) confirmed a strong clustering according to the ST, with the ST4034/t899 isolate differing from ST398/t899 by only 41 core alleles. Other characteristics, including matrix/sample origin, did not appear to cluster in this SNP analysis.

**TABLE 1:** Genotypic features of 34 methicillin-resistant Staphylococcus aureus *spa* type t899 isolates^*a*^

Accession no.	Isolate ID	Yr of isolation	Geographic origin	Isolate source	MLST	SCC*mec*	Tetracycline resistance gene
SRR1290901	28114	2013	Germany	Human	ST9	IVa(2B)	ND
SRR1290895	28144	2013	Germany	Poultry	ST9	ND	ND
SRR1303238	28194	2010	Netherlands	Human	ST9	IVa(2B)	*tet (L)*
SRR1290877	28212	—	Germany	Animal	ST9	IVa(2B)	*tet (L)*
SRR1303551	29908	—	Germany	Poultry	ST9	IVa(2B)	*tet (L)*
SRR1303423	29933	2010	Netherlands	Human	ST9	IVa(2B)	*tet (L)*
SRR1303430	30017	2011	Netherlands	Human	ST9	IVa(2B)	ND
ERR594184	30893	2014	Germany	Human	ST9	IVa(2B)	ND
SRR7825591	SAV1035	2017	Poland	Poultry meat	ST9	IVa(2B)	ND
SRR7825590	SAV1149	2017	Germany	Poultry meat	ST9	IVa(2B)	ND
SRR7825587	SAV1150	2017	Germany	Poultry meat	ST9	IVa(2B)	ND
SRR7825588	SAV1158	2017	Germany	Poultry meat	ST9	IVa(2B)	ND
SRR7825595	SAV1228	2017	Czech Republic	Pork	ST9	IVa(2B)	ND
ERR2442746	APHA06	2016	England	Turkey	ST398	IVa(2B)	*tet*(M)
ERR2562460	PHE3997901	2017	Scotland	Pheasant	ST398	IVa(2B)	*tet*(M)
SRR1218618	10S00488	2010	Germany	Turkey meat	ST398*	IVa(2B)	*tet*(M)
SRR1290866	81070	—	Germany	Human	ST398	IVa(2B)	*tet*(M)
SRR1290867	11S01586	2011	Germany	Camembert	ST398*	IVa(2B)	*tet*(M)
SRR1290868	12-ST01988	2012	Germany	Turkey meat	ST398*	IVa(2B)	*tet*(M)
SRR1290875	12S01399	2012	Germany	Turkey meat	ST398*	IVa(2B)	*tet*(M), *tet*(K)
SRR1300909	63-D10	2012	France	Turkey	ST398*	IVa(2B)	*tet*(M)
SRR1303281	12S00881	2012	Germany	Turkey meat	ST398*	IVa(2B)	*tet*(M)
SRR1303432	2010-60-7626-19	2010	Denmark	Chicken meat	ST398*	IVa(2B)	*tet*(M)
SRR1303468	12S01153	2012	Germany	Turkey meat	ST398*	IVa(2B)	*tet*(M)
SRR1303550	81629	—	Denmark	Human	ST398	IVa(2B)	*tet*(M)
SRR1303558	10S01493	2010	Germany	Turkey meat	ST398*	IVa(2B)	*tet*(M)
SRR445027	12152-5	2008	Italy	Pig	ST398*	IVa(2B)	*tet*(M)
SRR445029	30116	2008	Italy	Pig	ST398*	V(5C2&5)	*tet*(M)
SRR445030	44523-1	2008	Italy	Pig	ST398*	IVa(2B)	*tet*(M)
SRR445060	UB08116	2008	France	Pig (dust)	ST398	IVa(2B)	*tet*(M)
SRR7825589	SAV1146	2017	Germany	Poultry meat	ST398	IVa(2B)	*tet*(M)
SRR7825592	SAV1109	2017	Poland	Poultry meat	ST398	IVa(2B)	*tet*(M)
SRR7825593	SAV0154	2013	Czech Republic	Pork	ST4034	IVa(2B)	*tet*(M)
SRR7825594	SAV0987	2017	Czech Republic	Human	ST398	IVa(2B)	*tet*(M)

a *, CC398/CC9 hybrid strain; *tet*, tetracycline resistance gene; —, not available; ND, not detected. Isolates with SAV numbers are unique to this study.

The phylogenetic tree (Fig. 2) also showed major divergences in the antimicrobial resistance and virulence patterns depending on the ST. All ST398 isolates carried the *tet*(M) gene, while none of the ST9 isolates carried this tetracycline resistance gene (Fig. 2). Concerning virulence markers, most ST398 *spa* type t899 isolates harbored the *scn* and *sak* genes, indicating the presence of the IEC cluster, while ST9 isolates were devoid of the IEC cluster but systematically harbored the *seg*, *sei*, *sem*, *sen*, and *seu* genes, encoding enterotoxin-like proteins (Fig. 2).

## DISCUSSION

In this study, the SNP-based phylogeny analysis was consistent with the core genome multilocus sequence typing (cgMLST) analysis, with t899 isolates clustering apart based on STs. Although *spa* typing has a remarkable predictive power over clonal relationships, predicting genetic relatedness based on *spa* type does not appear appropriate for isolates that have undergone major recombination events, including *spa* gene passages (26–31). This important genomic recombination is not frequent in S. aureus, and the major representatives of such events are ST239, ST34, and ST42. The CC9/CC398 hybrid is another important example, giving rise to t899 isolates which largely diverge from their original CC9 genetic backgrounds and which mediate human diseases given their arsenal of virulence factors.

Here, we characterized t899 isolates from different STs using whole-genome-sequencing (WGS)-based approaches, together with epidemiological data, antimicrobial resistance genes, and virulence markers. This analysis revealed the differential occurrence of genes that can be used to further characterize t899 isolates. ST398-t899 isolates harbored the IEC cluster, which is crucial for disrupting the normal function of the human immune system (22, 32–34). Among the 34 t899 isolates tested, all ST398 representatives harbored the *tet*(M) gene, which is either transposon located or chromosomal, while ST9 representatives either were susceptible or carried the plasmid-located *tet*(L) gene. The tetracycline resistance gene *tet*(M) is a common feature of LA-MRSA ST398, while it is absent from MRSA ST9 (35–37). In contrast, ST9 isolates carried staphylococcal enterotoxin (SE) genes, which were not detected in ST398 isolates. This clear discrepancy between the two lineages would be useful to refine the LA-MRSA characterization when only *spa* typing is used and indicates the presence of t899 isolates.

Overall, investigations into S. aureus populations using WGS would be useful for future molecular epidemiology studies and for more closely examining the global evolution of S. aureus lineages. WGS also helps to assess the performances of classical typing methods by comparison. According to David et al. (38), two genotyping methods examining distinct genetic loci will not consistently provide identical results in classifying MRSA isolates, mostly because these methods assess genetic differences that can evolve independently. Classification systems often employed for epidemiological research have created competing nomenclatures that are useful for assessing the relatedness of isolates but are unfortunately not always directly comparable. This study emphasizes that *spa* typing is not sufficient to characterize t899-positive LA-MRSA. Accordingly, this study suggests the usefulness of an additional genomic marker to assign t899-positive MRSA isolates to the ST9 or ST398 clone, which may include *tet*(M), *sak*, and/or *seg* genes. Of course, this analysis should be refined when new t899 isolates belonging to other STs are sequenced and characterized.

## MATERIALS AND METHODS

### Bacterial collection.

Thirty-four t899 S. aureus isolates were found in the publicly available databases, and their corresponding characteristics (MLST, matrix [human, food, and animal origins], and geographical origin) were recorded. Raw reads were downloaded from NCBI, reads were quality checked with FastQC v.0.65, and low-quality reads were trimmed using Trimmomatic v.0.36.4 (39). Subsequently, contigs were generated using the SPAdes ve.3.5.0 algorithm (40), and those whose length exceeded 200 bp were retained in the assembly. In the literature, *spa* type t899 was also found to belong to 15 other SLVs and multilocus variants (MLVs) of ST9 and ST398 (see Table S1 in the supplemental material). These isolates could unfortunately not be included in our analysis because of the absence of associated WGS data.

### cgMLST analyses.

Isolates were subjected to cgMLST analyses. Genome-wide gene-by-gene microbial typing was performed using Ridom SeqSphere+ S. aureus cgMLST analysis with default parameters (41). The cgMLST data contain 1,861 coding loci representing the core genome (41). Once an allelic profile was assigned to each genome, a minimum spanning tree was constructed from the concatenated core genome sequences and visualized using the online tool PHYLOViZ. cgMLST loci with no allele calls were ignored in the pairwise comparison during the tree construction. The minimum spanning tree constructed on the basis of cgMLST data illustrates clusters by ST, *spa* type, or matrix (Fig. 1).

### SNP analysis and phylogenetic tree.

A phylogenetic tree was constructed based on single nucleotide polymorphism (SNP) analysis (9, 10, 22). SNPs were identified by mapping reads against the ST398 reference genome (strain S0385; GenBank accession no. AM990992). The maximum-likelihood phylogenetic tree was established in CSI Phylogeny using default settings (42). The phylogenetic tree visualization was realized using iTOL (Interactive Tree of Life) (43).

### Detection of resistance genes and selected virulence markers using WGS data.

The online tools ResFinder v.3.2 (44) and Virulence Finder v. 2.0 (45) from the Center for Genomic Epidemiology web-based platform were used to detect genes encoding potential resistance to antimicrobials and virulence markers, respectively. For a hit to be reported by the two programs, it had to cover at least 60% of the length of the gene sequence in the database with sequence identities of 60% and 90%, respectively. WGS-assembled data were used to perform the analysis.

### Data availability.

The sequence information for isolates SAV1035, SAV1149, SAV1150, SAV1158, and SAV1228 has been deposited in the SRA database under study accession number SRP161670. Individual accession numbers are listed in Table 1.

## Supplementary Material

Supplemental file 1
