# Supplementary material for: Genomic Insights into Methicillin-Resistant Staphylococcus aureus spa Type t899 Isolates Belonging to Different Sequence Types
Source: Appl Environ Microbiol. 2021 Feb 26;87(6):e01994-20. doi: 10.1128/AEM.01994-20 (PMC8105008; doi:10.1128/AEM.01994-20)
Supplement: Supplemental file 1 [file AEM.01994-20-s0001.pdf]

Supplementary table 1: Methicillin-resistant and susceptible *Staphylococcus aureus* t899 isolates belonging to different sequence types

**Id**	**Isolate**	**Country**	**Year**	**Detailed Source**	**Methicillin**	***arcC***	***aroE***	***glpF***	***gmk***	***pta***	***tpi***	***yqiL***	**ST (MLST)**
3327	P_37	Switzerland	2008	pig nasal swab	R	3	3	1	1	1	1	10	9
2693	1110807020	China	2008	enviroment	R	3	73	1	1	1	1	10	1376
2358	NICPBP-ST-P65	China	2008	animal	R	3	203	1	1	1	1	10	1297
4924	PUMCH2128	China	2010	sputum	R	3	3	1	1	1	1	296	2593
557	G16	China	2013	swine	R	3	3	1	1	1	264	10	164
3541	M15	China	2016	pig fecal	R	3	3	1	1	1	218	10	1605
3603	SR70	Taiwan	2012	animal	R	3	3	1	279	1	1	10	3319
1785	MRSA-C2487	China	2005	sputum	R	3	3	126	1	1	1	10	968
32911	S7	China	2014	nasal swab	S	3	1	1	1	1	1	10	4292
2693	1110807020	China	2008	dust sample	R	3	73	1	1	1	1	10	1376
33898	2B1-1	China	2016	human nasal swab	-	307	3	1	295	1	1	10	5051
4235	XJ22	China	2009	mastitis	R	3	35	19	2	20	26	39	398
32410	SAV0154	Czech Republic	2013	pork meat	R	281	35	19	2	20	26	39	4034
31657	M38	China	2016	pork	R	151	3	215	34	175	180	169	3387
32912	S31	China	2014	animal	S	151	36	215	34	175	180	169	4293

Allelic profiles for the seven house keeping genes: carbamate kinase - ***arcC***, shikimate dehydrogenase - ***aroE***, glycerol kinase - ***glpF***, guanylate kinase - ***gmk***, phosphate acetyltransferase - ***pta***, triosephosphate isomerase - ***tpi*** and acetyl coenzyme A acetyltransferase - ***yqiL***. " - " – not available. Strains obtained from https://pubmlst.org/bigsdb?db=pubmlst_saureus_isolates&page=query
